# Protecting effect of PrP codons M142 and K222 in goats orally challenged with bovine spongiform encephalopathy prions

**DOI:** 10.1186/s13567-017-0455-0

**Published:** 2017-09-19

**Authors:** C. Fast, W. Goldmann, P. Berthon, K. Tauscher, O. Andréoletti, I. Lantier, C. Rossignol, A. Bossers, J. G. Jacobs, N. Hunter, M. H. Groschup, F. Lantier, J. P. M. Langeveld

**Affiliations:** 1 Friedrich-Loeffler-Institut, Institute of Novel and Emerging Infectious Diseases, Greifswald-Insel Riems, Germany; 2 The Roslin Institute and Royal (Dick) School of Veterinary Studies, University of Edinburgh, Easter Bush, Midlothian, UK; 3 UMR 1282 ISP, Institut National de la Recherche Agronomique (INRA), University of Tours, 37380 Nouzilly, France; 4 INRA, UMR 1225, Interactions Hôtes Agents Pathogènes, Ecole Nationale Vétérinaire de Toulouse, Toulouse Cedex, France; 5 Wageningen BioVeterinary Research, Wageningen University & Research, Houtribweg 39, 8221RA Lelystad, The Netherlands

## Abstract

Breeding towards genetic resistance to prion disease is effective in eliminating scrapie. In sheep, classical forms of scrapie have been eradicated almost completely in several countries by breeding programs using a prion protein (PrP) gene (*PRNP*) amino acid polymorphism. For goats, field and experimental studies have provided evidence for several amino acid polymorphisms that are associated with resistance to scrapie, but only limited data are available concerning the susceptibility of caprine *PRNP* genotypes to BSE. In this study, goat kids representing five *PRNP* genotypes based on three polymorphisms (M142, Q211 and K222 and the wild type I142, R211 and Q222) were orally challenged with bovine or goat BSE. Wild type goats were killed with clinical signs between 24–28 months post inoculation (mpi) to both challenges, and goats with genotype R/Q211 succumbed between 29–36 mpi. I/M142 goats developed clinical signs at 44–45 mpi and M/M142 goats remained healthy until euthanasia at 48 mpi. None of the Q/K222 goats showed definite clinical signs. Taken together the highest attack ratios were seen in wild type and R/Q211 goats, and the lowest in I/M142, M/M142 and Q/K222. In all genotype groups, one or more goats remained healthy within the incubation period in both challenges and without detectable PrP deposition in the tissues. Our data show that both the K222 and M142 polymorphisms lengthen the incubation period significantly compared to wild type animals, but only K222 was associated with a significant increase in resistance to BSE infection after oral exposure to both BSE sources.

**Electronic supplementary material:**

The online version of this article (doi:10.1186/s13567-017-0455-0) contains supplementary material, which is available to authorized users.

## Introduction

Prion diseases or transmissible spongiform encephalopathies (TSEs) are caused by a unique infectious agent (“prion”) characterised by an entirely proteinaceous nature with apparent absence of functional nucleic acids [1, 2]. Disease transmission is possible within and between mammalian host species. Pivotal for transmissibility is the host’s prion protein (PrP) that in its normal state is a cell membrane protein (PrP^C^) to which recently several potential functions have been ascribed either in receptor mediation or immunological quiescence [3, 4]. In prion diseases PrP^C^ is converted into a stable “pathological” conformer (PrP^Sc^), which is the diagnostic disease marker detectable in immunohistochemistry by its disease associated deposition patterns (PrP^D^) and biochemically by its aggregation properties and protease resistance of its C-terminal core region (PrP^res^) [5–7].

The emergence of bovine spongiform encephalopathy (BSE) in cattle and its subsequent transmission to humans as variant Creutzfeld–Jakob disease (vCJD) has proven that prion diseases represent a threat for man and other mammalian species [8, 9]. Goats represent the only other domestic ruminant species to be affected by BSE under field conditions [10–12]. Furthermore, goats are susceptible to classical scrapie although the disease occurs in Europe generally at 2–3 times lower prevalence than in sheep [13]. Susceptibility to TSE infection in sheep and goats has a strong genetic component that potentially allows selection by breeding for resistance. The basis for this selection is the polymorphic character of the PrP amino acid sequence, because some of these PrP variants do not convert easily to PrP^Sc^ [14]. Breeding towards resistance has been successfully accomplished for sheep in some EU countries using the Q171R amino acid polymorphism as selection target, helped by the fact that the R171 allele appears to be dominant with almost complete protection to natural scrapie in R/R171 homozygotes as well as in Q/R171 heterozygotes [13, 15, 16].

A review of genotype surveys conducted worldwide revealed that on average almost 40% of goat *PRNP* sequences showed some variation of the amino acid sequence compared to the wild type [17, 18]. Case control studies have narrowed the potential candidates for resistance-associated polymorphisms to six amino acid changes: M142, S146, D146, H154, Q211 and K222 (wild type I142, N146, R154, R211 and Q222) [19–25]. It should be noted that wild type and variant *PRNP* alleles vary in codon 240 either encoding S240 or P240. While the wild type allele appears to split on average equally between S240 and P240 sequences, some of the variant alleles have so far only been observed in one combination, for example K222-S240. Association of codon 240 with TSE resistance is most likely weak or absent [19–21, 26]. The occurrence and frequencies of these *PRNP* alleles are breed and region dependent [27–29]. The strong genetic resistance to classical scrapie conferred by the K222 allele was corroborated by studies in goats using intracerebral and oral scrapie challenges with isolates from Italy, the USA or France [30–32].

We have previously published an interim report on a study of an oral challenge in goats with caprine BSE [33]. Here we report the final and full results of this study in which caprine and bovine BSE isolates were inoculated orally into goats of five different genotypes which were homozygous wild type (wt/wt) and M/M142 or heterozygous I/M142, R/Q211 and Q/K222. The data provide convincing evidence that the K222 allele is the strongest protective factor in goats against BSE.

## Materials and methods

### Animals

Three different laboratories were involved in the challenges to exploit the available space: INRA Nouzilly, France (lab1), The Roslin Institute, UK (lab2) and FLI, Germany (lab3). Animal experimentation was performed according to European directive 2010/63/EU as well as in compliance with the respective national legislations in these countries (reference number for Germany LALLF 7221.3-2.5-001/05, Animal & Scientific Procedures Act 1986, UK). At INRA (France), all experiments were conducted in accordance with the guidelines of the European Council Directive (86/609) and approved by the local ethical committee; the animals were kept in Biosafety Level 3 confined housing (PFIE, UE-1277, INRA Centre Val de Loire, Nouzilly, France). Goat kids of different breeds were obtained after weaning from the following sources: for lab1 and lab3 through Francis Barillet (goat production at INRA Centre Val-de-Loire in Bourges Experimental Unit, INRA division of Animal Genetics) and for lab2 through acquisition from UK holdings and through artificial insemination of females from the Roslin Institute. The animals belonged to the following breeds: Alpine, Saanen, Boer, Toggenburg and their crosses. Animals had been bred and selected based on their *PRNP* genotype, they were either I/M142, M/M142, R/Q211, Q/K222 or homozygous wild type for any of these codons (wt/wt). In total 129 animals were used in this study (Table 1).

**Table 1:**
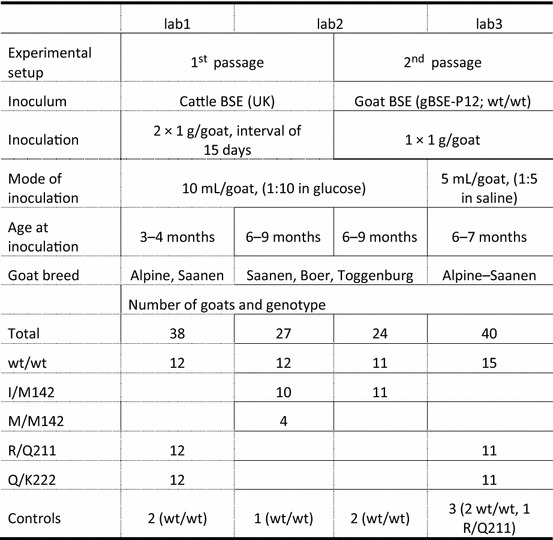
**Experimental setup in the different labs performing the goat oral challenge**

BSE, bovine spongiform encephalopathy; UK, United Kingdom; gBSE-P12, identification number of goat brain pooled homogenate; wt/wt, homozygous wild type; I/M142, heterozygous isoleucine/methionine genotype at codon 142; M/M142, homozygous methionine genotype at codon 142; R/Q211, heterozygous arginine/glutamine genotype at codon 211; Q/K222, heterozygous glutamine/lysine genotype at codon 222.

### Challenge and disease monitoring

Bovine and goat BSE brain materials were derived from clinically and PrP^Sc^—positive confirmed cases. The cattle BSE homogenate for first passage was derived from a pool of two clinically affected UK BSE cases supplied by the VLA (now APHA, Weybridge, UK) (Table 1). The goat BSE homogenate for second passage was pooled brain material derived from three clinically and PrP^Sc^ positive animals of wt/wt genotype following intracerebral challenge with cattle BSE [34]. Animals were orally challenged twice with 1 g of bovine BSE in 10 mL physiological saline solution with a 2 weeks interval (lab1, lab2) or only once with 1 g of goat BSE in 5 mL saline due to the lower amount of goat brain material available (lab2, lab3). During the incubation period, blood samples (lab1–3) as well as tonsil (lab1–3) and rectal (lab3) biopsies were taken on a regular scheme (Additional file 1). One to three animals per genotype were euthanized at first at pre-defined time points during the observation period. In addition goats were killed after showing consistently clinical signs typical for BSE (i.e. neurological disorders such as abnormalities in sensation and movement) or due to animal welfare reasons other than BSE. The last goats were killed due to animal welfare reasons other than BSE at 81 mpi. Seven unchallenged wt/wt goats (lab1 two, lab2 three, and lab3 two) and one unchallenged R/Q211 (lab3) shared the pens with challenged animals between 13 and 47 months of the inoculation period to control for horizontal transmission. At necropsy a wide range of tissue samples were harvested under TSE-sterile conditions.

### Antibody sources

PrP specific antibodies used and their sources were: Bar224, Sha31 and SAF84 [35], 6C2 and 12B2 [36, 37], R145 [38], F99/97.6.1 [39], P4 and L42 [40], and 6H4 [41]. R145 and F99/97.6.1 were epitope mapped by Pepscan analysis to the respective ovPrP sequences 223RESQ226 and 221YQRE224 following published methods [42].

### Histology and immunohistochemistry

The formalin fixed tissue samples were hematoxylin and eosin (H&E) stained and immunohistochemically (IHC) processed with well established procedures using PrP specific antibodies [38, 43, 44]. Methodological details are summarized in Additional file 1.

### Biochemical analysis for scrapie-BSE discrimination and in-depth TSE typing

Brain stem samples of all animals were further examined by Western blot for discrimination between classical scrapie and BSE (Additional file 1) as described before [44–47]. Furthermore for in-depth TSE typing, triplex Western blot (triplex-WB) analyses were carried out as described before, using a mix of three antibodies, 12B2, Sha31 and either SAF84 or F99 after first immuno-complexing these respectively with Zenon labels Alexa 647, 555, and 488 [48]. These antibodies are markers: (1) for presence of the N-terminal epitope (12B2) typical for classical scrapie but absent in classical BSE, and CH1641 scrapie, (2) the core region of PrP^res^ (Sha31), and (3) the presence of a second PrP^res^ population covering the C-terminal PrP region between the Sha31 epitope (sequence 148YEDRYYRE155, ovine numbering) and the C-terminus of mature PrP which is only recognized by antibodies like SAF84 and F99 (respective epitope sequences 166YRPVDQY171 and 221YQRE224) and which fragment is characteristic for CH1641 scrapie and H-type BSE. For comparison, reference samples used were: C-type, H-type and L-type BSE from cattle, and small ruminant BSE, classical scrapie, and CH1641 scrapie (sheep and/or goat) were used as in previous publications [31, 49].

### Mouse bioassay

In lab1 and lab3 mouse bioassays were performed at 6 and 12 mpi with different tissues taken from respectively cattle BSE and goat BSE infected goats to test for the eventual presence of infectivity in the early stage of disease incubation. In doing so transgenic mice overexpressing ovine (in lab1 Tg338 and in lab3 TgshpIX) and bovine PrP (in lab 1 Tg110) were used. All mouse lines are known to be highly sensitive for the detection of prion infectivity of different origin including BSE (Tg110 in [33], the Tg338 and Tgshp IX in preparation by Nonno et al.). Additionally end-point titration experiments with a goat BSE isolate were done using Tg110 [33] and TgshpIX mice (lab 3, unpublished results), but data are not included here. The mice were intracerebrally inoculated (6–15 mice per sample, depending of the different lab schedules routinely done in France and Germany) with 20–30 µL of a 10% tissue homogenate diluted in physiological saline [45, 50, 51]. Subsequently all mice were clinically checked at least twice a week and either sacrificed due to animal welfare reasons or after 730 dpi at the latest. The mouse brains were subsequently tested for the presence of proteinase K resistant PrP^Sc^ (PrP^res^) by Western blot [44].

### Statistical analysis

Differences in incubation period lengths were analysed for statistical significance with Student’s T test and significance for differences in distribution between healthy and disease animals by challenge and genotype groups were analysed with Fisher’s Exact Test for a 2 × 2 Contingency Table with two-tailed probability (p).

## Results

Goat challenge results were classified in four health stage categories: (a) clinical—when consistent neurological signs pointing to a BSE infection were observed, (b) late preclinical—when no obvious clinical signs were observed but the animal appeared post mortem positive by IHC in the central nervous system (CNS), (c) early preclinical—when animals showed positivity by IHC in peripheral tissues but not yet in the CNS, and (d) healthy—without any of these markers. The occurrence of these categories in the time line after challenge of individual goats with their genotypes can be viewed in Additional file 2. Additionally, the time points mentioned below refer to the date of necropsy and subsequent prove of BSE infection by the detection of PrP^D^ in the brain stem. However, it has to be born in mind that some of these goats displayed the first clinical signs up to 6–12 weeks before necropsy.

### Goat challenges: first passage (bovine BSE)

**Figure 1:**
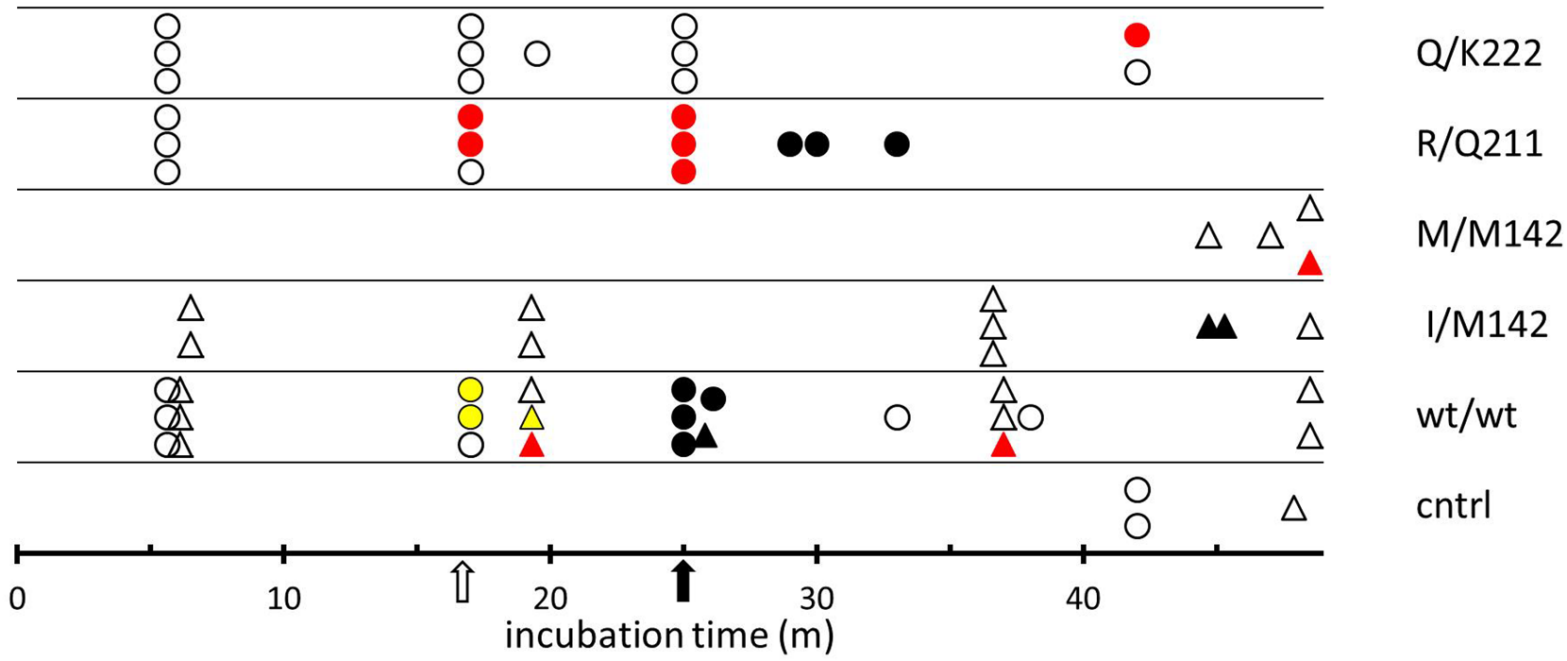
**Overview of the results of challenge with bovine BSE (1** ^**st**^ **passage) in goats with five different genotypes. **Each symbol represents an individual goat. Symbol colours: empty, yellow, red and black represents TSE negative, preclinical PrP^D^ (positivity in peripheral sites), late preclinical (PrP^D^ positivity in CNS, no clear clinical signs) and clinical cases (these are also PrP^D^ positive in periphery and CNS). Symbol shapes represent location of experiment: circles, lab 1, triangles lab 2 (see also “Materials and methods” section). The two arrows under the X-axis: open and bold indicate respectively the month after inoculation at which respectively the first preclinical and clinical cases were observed.

Sixty-two animals were challenged with bovine BSE in lab1 and lab2. The goats were in five *PRNP* genotype groups: wt/wt (n = 24), I/M142 (n = 10), M/M142 (n = 4), R/Q211 (n = 12) and Q/K222 (n = 12). Three unchallenged wild type animals were kept as contact controls (Table 1). In total 22 animals were affected by BSE, while 40 goats and the controls remained completely negative with observation periods up to 48 months post-inoculation (mpi) (Figure 1).

Clinical signs appeared in ten goats: five wt/wt goats at 25/26 mpi, three R/Q211 animals between 28–33 mpi and even later at 44/45 mpi in two I/M142 goats. All clinically affected animals were PrP^D^ positive by immunohistochemistry (IHC) in the central nervous system (CNS) and peripheral tissues in various combinations (see below).

A further nine animals were in a late preclinical state at necropsy and revealed PrP^D^ in brain stem as shown by IHC and/or biochemical analysis, but none of these goats showed clearly identifiable clinical signs typical for BSE. This group included two wt/wt goats sacrificed at 19 and 36 mpi, five R/Q211 goats sacrificed at 17 (n = 2) and 25 (n = 3) mpi, one Q/K222 goat killed at 43 mpi and one M/M142 goat killed at 48 mpi.

Another three wt/wt goats were in an early preclinical state at 17 (n = 2) and 19 (n = 1), revealing weak amounts of PrP^D^ in different peripheral tissues only (see below). Samples from CNS and spleen from nine (3 wt/wt, 3 R/Q211, 3 Q/K222) healthy, BSE negative goats, which were culled at 6 mpi as part of the pathogenesis study, were examined by mouse bioassay using transgenic mouse lines Tg110 and Tg338. None of these mice showed clinical or pathological signs of a TSE infection.

The 40 non-affected animals were euthanized at various time points with the longest survival times in the different genotypes as follows: 48 mpi for 2 wt/wt, one I/M142 and one M/M142, and 43 mpi for one Q/K222 goat.

Because some animals were removed from the challenge groups at pre-defined post-inoculation times, attack ratios for preclinical, late preclinical and clinical animals 12 mpi and later were only considered for estimation. These ratios were not less than 56% in wt/wt, 25% in I/M142 goats, 25% in M/M142, 89% in R/Q211, and 11% in Q/K222 goats (Table 2). These values decreased for four genotypes when only clinical positive animals were included: 28% in wt/wt, 0% in M/M142, 33% in R/Q211 and 0% in Q/K222 goats.

**Table 2:** **Data concerning the effectiveness of the oral challenges in goats with bovine (1** ^**st**^ **passage) and caprine (2** ^**nd**^ **passage) BSE**

Genotype	Numbers			Numbers from 12 mpi				Inc. time clinical, mpi ±SD (n)
	Total	Before 12 mpi	After 12 mpi	Pre-clinical	Late preclinical	Clinical	Attack ratios affected/total (%) + significance^a^	
bovBSE								
wt	24	6	18	3	2	5	10/18 (56%)	25.2 ± 0.4 (5)
I/M142	10	2	8	0	0	2	2/8 (25%)**	44.5 ± 0.7 (2)
M/M142	4	0	4	0	1	0	1/4 (25%)	NA
R/Q211	12	3	9	0	5	3	8/9 (89%)	30.7 ± 2.1 (3)
Q/K222	12	3	9	0	1^b^	0	1/9 (11%)**	NA
gtBSE								
wt	26	6	20	2	3	5	10/20 (50%)	25.6 ± 1.5 (5)
I/M142	11	3	8	0	0	0	0/8 (0%)*	NA
R/Q211	11	4	7	1	0	3	4/7 (57%)	34.3 ± 1.5 (3)
Q/K222	11	3	8	0	1^c^	0	1/8 (13%)^c^*	NA

Genotype	Total	mpi			Numbers from 12 mpi			Inc. time clinical, mpi ±SD (n)
		1^st^ clin case	Last clin case	Pre-clinical	Late preclinical	Clinical	Attack ratios affected/total (%) + significance	
Combined challenges								
wt	50	24	28	5	5	10	20/38 (53%)	25.4 ± 1.1 (10)
I/M142	21	44	45	0	0	2	2/16 (13%)**	44.5 ± 0.7 (2)
M/M142	4	NA	NA	NA	NA	NA	NA	NA
R/Q211	23	28	36	1	5	6	12/16 (75%)	32.5 ± 2.6 (6)
Q/K222	23	43	NA	0	2	0	2/17 (12%)***	NA

NA, not applicable.

^a^Statistical data: compared to the wild type group. Fisher’s exact test, *, **, and *** respectively *p* < 0.05, *p* < 0.01, *p* < 0.001.

^b^This Q/K222 goat was CNS- and muscle-positive.

^c^One Q/K222 case was CNS-positive, and psoas-muscle-positive but by mouse bioassay (Tg110) only [33]. If considered negative (by IHC) this group was statistically positive compared to the wild type group (*p* < 0.05).

### Goat challenges: second passage (goat BSE)

**Figure 2:**
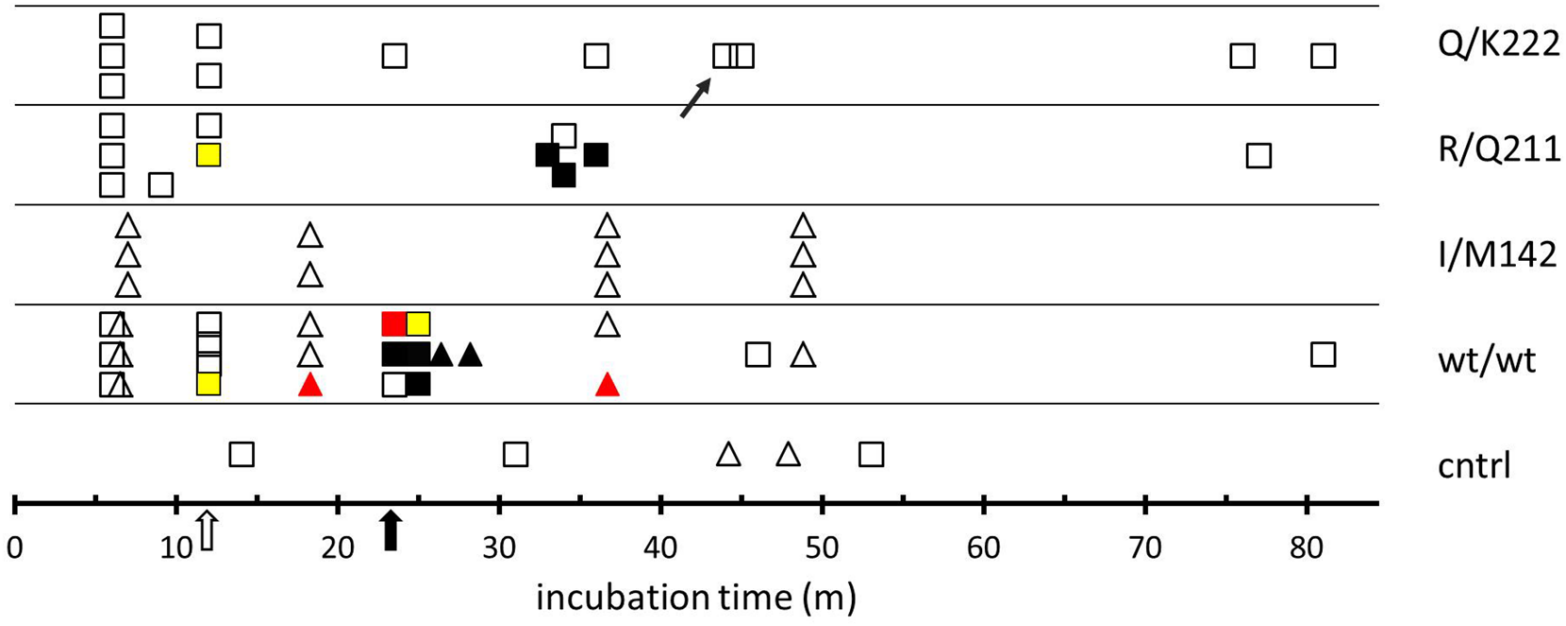
**Overview of the results of challenge with goat BSE (= 2** ^**nd**^ **passage) in goats with four different genotypes.** Each symbol represents an individual goat. Symbol colours and arrows are explained in Figure 1. Symbol shapes represent the laboratory of experiment: triangles lab 2, and squares lab 3. The two arrows under the X-axis: open and bold indicate respectively the month after inoculation at which respectively the first preclinical and clinical cases were observed. Small arrow near one Q/K222 symbol is reflecting the animal previously found weakly brain (only) positive in infectivity testing in Tg110 mice [33].

Fifty-nine goats were challenged with goat BSE in lab 2 and lab 3. They were in four *PRNP* genotype groups: wt/wt (n = 26), I/M142 (n = 11), R/Q211 (n = 11), and Q/K222 (n = 11) (Table 1). Four wildtype and one R/Q211 animals were kept as unchallenged contact controls. In total 15 animals were affected by a BSE infection, and 44 goats and the controls remained completely negative within the observation period up to 81 mpi (Figure 2).

Clinical signs appeared in eight goats: five wt/wt goats between 24 and 28 mpi, and three R/Q211 animals between 33–36 mpi. All clinically affected animals were PrP^D^ positive by IHC in the CNS and in peripheral tissues in various combinations (for details see below).

Additional four animals were in a late preclinical state at necropsy revealing PrP^D^ or infectivity in brain stem as shown by IHC. None of these goats showed clinical signs typical for BSE. This group included three wt/wt goats at 19, 24 and 36 mpi as well as one Q/K222 goat at 45 mpi (infectivity data from this goat are published in [33]).

Two wt/wt goats (12 and 25 mpi) and one R/Q211 goat (12 mpi) were in an early preclinical state, revealing weak amounts of PrP^D^ in peripheral tissues only (for details see below). None of the I/M142 and Q/K222 goats showed clinical TSE signs.

Various samples from CNS (brain stem) and peripheral tissues (including samples from gut, lymphoreticular tissues as well as autonomous nervous system) from two healthy and BSE negative wt/wt goats, which were culled at 12 mpi as part of the pathogenesis study, were examined by mouse bioassay using transgenic mouse lines TgshpIX. None of these mice showed clinical or pathological signs of a TSE infection.

The 44 non-affected animals were euthanized at various time points with the longest survival times in the different genotypes as follows: 81 mpi for one wt/wt and one Q/K222 goat, 47 mpi for three I/M142 goats and 77 mpi for a R/Q211 goat.

Similar to the first passage BSE study, challenged animals 12 mpi and later were only considered for estimation of attack ratios. These attack ratios for preclinical, late preclinical and clinical animals together were not less than 50% in wt/wt, 0% in I/M142 goats, 57% in R/Q211, and 13% in Q/K222 goats (Table 2). Including only clinically positive animals reduced these values to 25% in wt/wt, 43% in R/Q211 and 0% in Q/K222 goats, while I/M142 goats remained unchanged.

### Statistical analysis

There was no statistically significant difference in the wild type challenge outcomes regarding the number of affected animals or the incubation period length of clinically affected goats between the two BSE isolates or between the three different laboratories. The small number of goats developing clinical disease made it difficult to analyse differences of incubation period length between genotypes.

Considering the period from 12 mpi, where the first preclinical signs appeared in the challenges (12mpi, in a wt and a R/Q211 goat), till the end of the experiments (see Figure 2) there were significant differences in attack ratios between wild type and QK222 groups in both challenges if only based on IHC observations (Table 2). The attack ratios values between wt and IM142 goats in goat BSE challenge were also statistically different (p < 0.05), but not after challenge with bovine BSE.

Furthermore, differences became also apparent when the observation period was divided into early (12–30 mpi) and late (> 30 mpi) phase and when both challenges were combined. In the early phase, infected animals were found only in wt/wt and R/Q211 goats at ratios of 18/25 (72%) and 7/11 (64%), respectively; no infected animals occurred in genotypes I/M142 (0/4) and Q/K222 (0/10). The difference between group I (wt/wt, R/Q211) and group II (I/M142, Q/K222) was highly significant (*p* < 0.0001).

The late phase showed no significant difference between group I and group II and all genotypes had infected animals. After 30 mpi, R/Q211 had a higher attack ratio of 5/7 (71%) than either wt/wt with 2/12 (17%) or I/M142 with 2/12 (*p* = 0.03), but the difference to Q/K222 (2/7) was not significant. While the minimal attack ratio for R/Q211 was unchanged between the two observation phases, wt/wt genotypes were significantly less likely to be infected (*p* < 0.002) once they had survived 30 mpi.

### Immunohistochemistry

There were no distinct differences between the animals from the first and the second passage, therefore the immunohistochemical results will be presented for all groups together (see Additional files 3 and 4 for further details in respectively first and second passage results).

**Figure 3:**
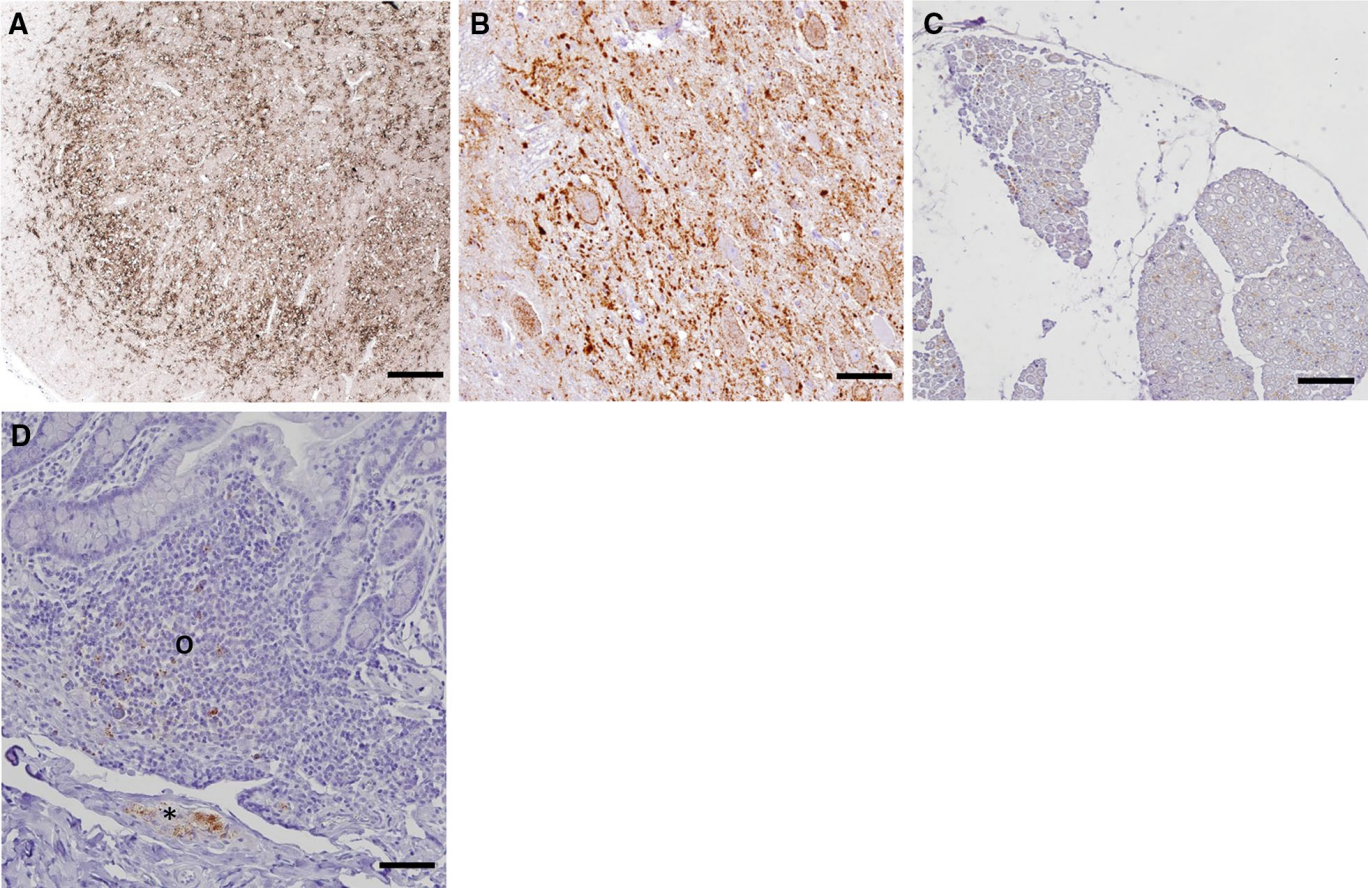
**PrP** ^**D**^ **accumulation in central and peripheral nervous tissues of BSE infected goats. A** Brain stem of a clinical goat BSE infected wt/wt goat, 24 mpi, showing a severe intra- and extracellular PrP^D^ accumulation. Antibody 6C2, bar 50 µm. **B** Brain stem of the late preclinical cattle BSE infected Q/K222 goat, 43 mpi, showing a severe intra- and extracellular PrP^D^ accumulation in the obex (DMNV). Antibody Bar224, bar 20 µm. **C** Spinal nerve of a clinical cattle BSE infected wt/wt goat, 25 mpi, showing moderate intra-axonal and intraglial PrP^D^ accumulation. Antibody Bar224, bar 100 µm. **D** Ileum of a clinical cattle BSE infected wt/wt goat, 25 mpi, showing PrP^D^ accumulation in neurons and glial cells of the enteric nervous system as well as intracellular in lymphoid cells of the ileal follicle. Antibody Bar224, bar 20 µm.

In total 30 goats revealed a clear PrP^D^ accumulation in the brain stem (CNS positive cases in Figure 3A). The degree of PrP^D^ deposition was associated with the clinical state of the animal. Thus, goats being in a late preclinical state showed a weak (n = 1), mild (n = 5) and moderate (n = 4) PrP^D^ accumulation; only one R/Q211 goat and one Q/K222 goat (Figure 3B) were severely affected. In contrast, animals with clear clinical signs mostly exhibited a severe PrP^D^ deposition (n = 16) and more rarely a moderate accumulation (n = 2). In most cases PrP^D^ accumulation was widespread and involved the whole brain stem, both grey and white matter. In animals with only mild PrP^D^ accumulation, the most prominent depositions were seen in the dorsal motor nucleus of the vagus nerve (DMNV), the nucleus of the solitary tract, cuneate nucleus, hypoglossal nucleus, spinal tract nucleus and the olivary nuclei. Using antibodies specific for the PrP C-terminus which detect BSE associated PrP^D^ very well [52], intraneuronal, intraglial and cell-membrane-associated/extracellular fine to coarse PrP^D^ accumulations were seen in all brain areas examined.

**Figure 4:**
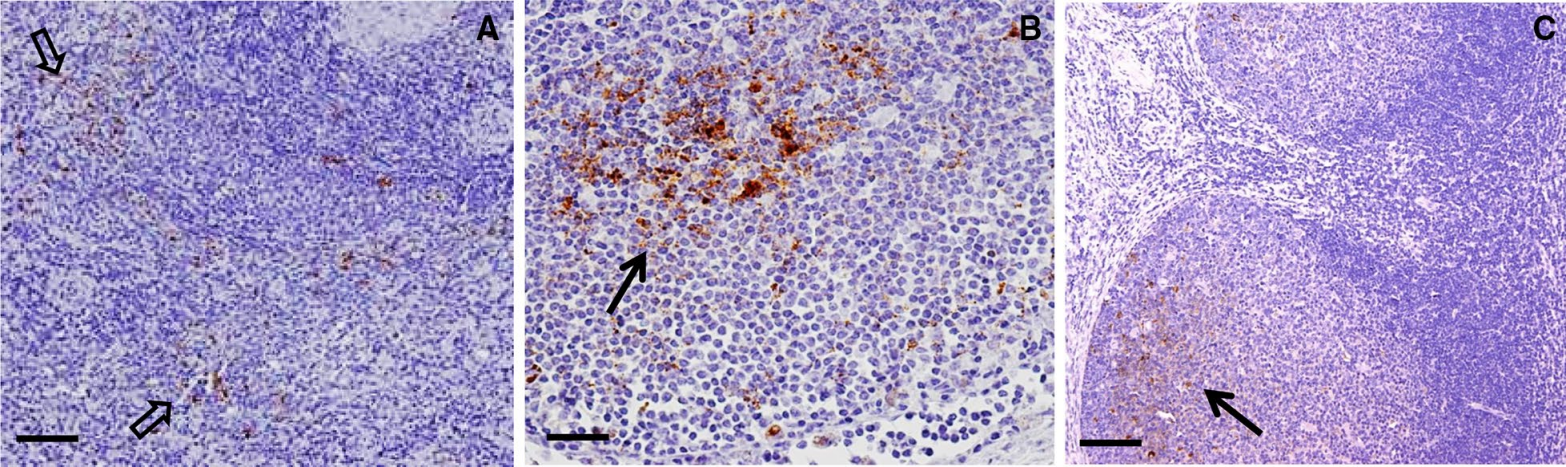
**PrP** ^**D**^ **accumulation in lymphoreticular tissues of BSE infected goats. A** Spleen of a clinical cattle BSE infected wt/wt goat, 26 mpi. Antibody Bar224, bar 50 µm. **B** Mesenterial lymph node of a clinical cattle BSE infected R/Q211 goat, 30 mpi. Antibody bar 224, bar 20 µm. **C** Tonsil of a clinical cattle BSE infected I/M142 goat. Antibody R145, bar 50 µm. In all samples a mild to moderate PrP^D^ accumulation in follicles and in the sinus of the spleen can be seen.

Several peripheral lymphoreticular tissue samples were analysed for PrP^D^ accumulation, amongst them were mesenterial lymph nodes (MesLn), tonsil, ileum including Peyer’s patches (PP), rectal follicles (RAMALT), celiac and mesenteric ganglion complex (CMGC), ileal and rectal enteric nervous system (ENS), vagal nerve and brachial plexus, along with different skeletal muscles (Mm. oculomotorius, psoas major and semitendinosus) (Figure 4). The analysis of five wt/wt and one R/Q211 goat, which were negative in the brain stem, revealed PrP^D^ accumulations in the tonsil (n = 1), CMGC (n = 2) and ENS of the Ileum (n = 2) and in the ileal PP (n = 1).

The ileal ENS was the most frequently positive site (n = 8) in the twelve late preclinical goats, which had only mild to moderate amounts of PrP^D^ in the brain stem. Only four animals showed a more widespread distribution of PrP^D^ involving the rectal ENS (n = 1), MesLn (n = 2), ileal PP (n = 2) and tonsil (n = 2).

**Figure 5:**
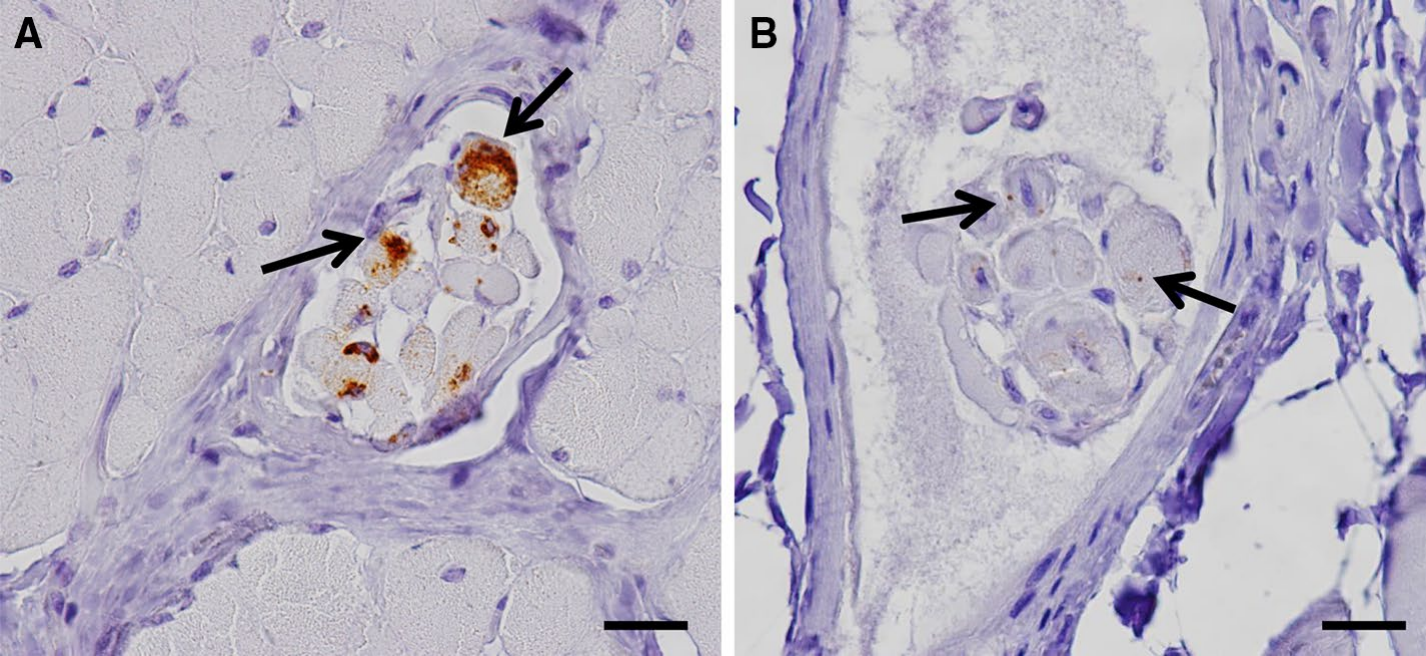
**PrP** ^**D**^ **accumulation in different skeletal muscles. A** Ocular muscle of a Q/R211 goat infected with cattle BSE showing PrP^D^ accumulation in muscle spindles, 28 mpi. Antibody Bar224, bar 20 µm. **B** Psoas muscle of a Q/K222 goat infected with cattle BSE showing weak PrP^D^ accumulation in muscle spindles, 43 mpi. Antibody Bar224, bar 20 µm.

The positive Q/K222 goat (43 mpi) which was in a late preclinical state, showed a severe PrP^D^ accumulation in the brain, but no involvement of the LRS (Figure 5). The only peripheral tissues slightly positive are rectal ENS and different muscles (M. oculomotorius, M. psoas major).

Clinically ill wt/wt and R/Q211 goats (n = 16) with moderate up to severe PrP^D^ accumulation in the brain stem, revealed a more widespread distribution of PrP^D^ in the LRS, ENS and different skeletal muscles with only five animals, in which PrP^D^ accumulations were confined to the ENS and PP of the gut. Two animals even showed a positive reaction in peripheral nerves. More interestingly are the two I/M142 goats, both with severely affected brains. PrP^D^ could be found in ileal ENS but not in the PP. Yet, one of these goats showed a mild PrP^D^ deposition in tonsil follicles.

In tissues of the LRS the PrP^D^ depositions were mostly confined to the follicles and rarely seen in the sinus. Reaction pattern seen is an intracellular fine to coarse granular staining in tingible body macrophages, follicular dendritic cells and few lymphocytes. Neuronal as well as glial cells were positive in the ENS (Figure 3), but in the CMGC only single neuronal cells showed a diffuse intracellular pattern. Single muscle spindles revealed a mild accumulation of PrP^D^ (Figure 5).

### Discriminatory analysis and TSE typing

**Figure 6:**
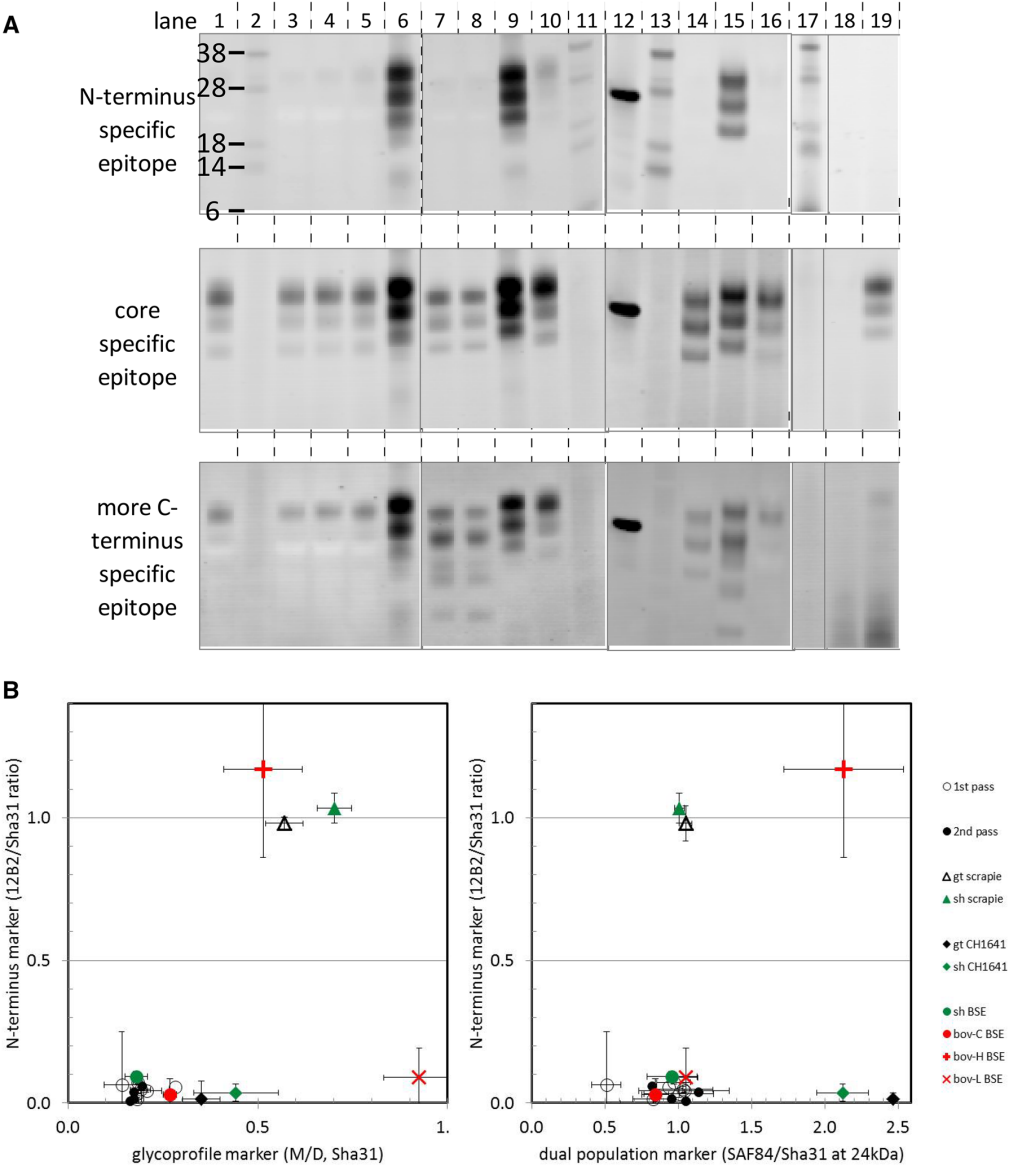
**Western blot: triplex Western blot analysis of goat BSE brain stem samples with three antibodies on one membrane.** Samples from orally challenged goats were analysed together with a set of different types of TSEs from goat, sheep and cattle to illustrate the classical BSE like character in the BSE-challenged goats. **A** Analysis with a mixture of three antibodies with different PrP specificities: N-terminus (12B2), core (Sha31) and C-terminus (SAF84, lanes 1–16; or F99, lanes 17–19). Lanes: 1, 3–5, 19 goat BSE material from respectively wt/wt 2^nd^ pass, R/Q211 2^nd^ pass, wt/wt 2^nd^ pass, R/Q211 2^nd^ pass, Q/K222 1^st^ pass; lane 6, classical scrapie from i.c. challenged R/Q211 goat; lane 7, CH1641 scrapie from i.c. challenge in wt/wt sheep; lane 8 CH1641 scrapie from i.c. challenge in wt/wt goat; lane 9 natural scrapie from wt/wt sheep; lane 10 BSE from i.c. challenged wt sheep 1^st^ pass; lanes 14–16 respectively bovine C-type BSE, H-type BSE and L type BSE; lane 18, non-challenged goat material. Applied tissue equivalents were 0.5 mg, or (in lanes 3, 4, 14–16 and 19) 1 mg. Lanes 2, 11, 13, and 17, mol mass markers are indicated with their kDa; in lane 12, 15 ng rec-ovine PrP (wt). For linear epitope specificities of the antibodies, see paragraph Biochemical analysis of the Methods section. **B** Dot plots showing the TSE-type markers of individual samples derived from the antibody signals of PrP^res^ in triplex-WB. Each symbol represents the average signal ratio obtained from a triplicate analysis per individual sample (bars for the standard deviations). All orally challenged goats yield a typical C-BSE pattern for the markers N-terminus, glycoprofile and dual population independent of genotype and passage (1^st^ or 2^nd^). The 12 analysed goat samples from the BSE challenges were: from 1^st^ pass two wt/wt, two I/M142, three R/Q211, and one Q/K222 cases (open circles); from 2^nd^ pass three wt/wt, and one R/Q211 cases (closed black circles).

Positivity in brain stem of all goats was analysed in the three laboratories, each with their own methodology to discriminate C-type BSE from classical scrapie (data not shown). Furthermore, in samples from goats of all genotypes and both passage numbers the type of TSE was studied in more detail using as reference samples bovine C-type, H-type and L-type BSE, ovine and caprine BSE, classical scrapie, and CH1641 scrapie (Figure 6A). The low presence of 12B2 epitope (12B2/Sha31 signal ratio < 0.3), the glycoprofile expressed as the ratio between mono- and diglycosylated PrP^res^ (< 0.4, using glycoform fractions of total PrP^res^ in the Sha31 epitope detection) and the presence of only a single PrP^res^ population pointed out that all positive samples did show the character of a C-type BSE infection (Figure 6B) [48, 49].

## Discussion

This oral challenge study in goats elucidates the significant protection conferred by the Q/K222 prion protein genotype against bovine and goat BSE infection administered through the oral route. There was no clear clinical case with this genotype in 17 challenged animals, although it has to be considered that both late-preclinical Q/K222 goat from the bovine and caprine BSE challenges might have developed clinical disease at some later stage. I/M142 heterozygous genotypes appeared to provide partial resistance, with two clinically positive cases. Including the M/M142 late-preclinical case, M142 is also clearly associated with lengthened incubation periods in BSE similar to scrapie challenges [31].

While the R/Q211 genotyped goats exhibited extended incubation periods of the clinical cases by about 5–9 months compared to the wild type goats in the two BSE challenges, it appeared that there was also a difference in the susceptibility beyond 30 months post inoculation. In that late phase of the experiments, 2 out of 7 (29%) animals survived BSE challenge in the R/Q211 group only whereas in the wild type group 10 out of 12 (83%) animals showed no signs of BSE infection. Additional amino acid polymorphisms in *PRNP* can be excluded as reason for a survival difference between the two genotypes, therefore other genetic loci might be involved [53]. It is unlikely though to be solely due to breed or laboratory differences as discussed below.

This study has been conducted in three laboratories in parallel following similar protocols as much as possible, but with different breeds of different regional origins, and different age groups. Despite this set-up, which was logistically necessary to conduct a challenge study of 129 goat kids, significant differences were not observed between the laboratory groups as evidenced by a short mean incubation period and a narrow standard deviation (25.4 ± 1.1, Table 2) for the ten clinical cases of the wild type groups. This finding is supported by the fact that the tissue distribution did not reveal any remarkable deviations between the different laboratories.

The goat kids used here were aged 3–9 months at challenge, depending on the lab involved. Previous findings in sheep and goats suggest that to achieve the highest attack rates in oral challenge, administration before weaning was important, possibly due to the developmental stage of the Peyer’s patches in the intestine [34, 54–56]. Although the exact response to challenge for the early age range was not known for goat kids, challenge of the kids before weaning was planned but could not be implemented due to logistic problems in animal movements. Nevertheless, the mean attack rate of 53% for all wild type animals in this study was distinctly higher than the 21% reported before for BSE challenged lambs of the 3–6 month age range [54]. It might reflect a higher intrinsic susceptibility of goats for oral challenge than sheep, which may also explain that BSE has only been identified in this species in natural conditions [10–12]. Taken together the data do not imply large variation due to selection of various age groups and breeds, but we cannot completely rule out that the sensitivity in the challenges of the five genotypes was reduced through an age effect.

Considering the successful transmissions in the different genotypes, our data showed that clinical disease was appearing first in the wt/wt goats at 24–28 mpi with no differentiation between cattle BSE (first passage) and caprine BSE (second passage). This is rather surprising, as it has been shown previously that interspecies transmission of TSEs results in long incubation periods, which usually shorten on further passages [57]. In particular, this result seems to contradict previous observations in transgenic mice expressing bovine PrP (Tg110), in which sheep- or goat-passaged BSE would lead to shorter incubation times than bovine BSE [58, 59]. Differences between the two challenge conditions or titre of the challenge materials might have played a role. On the other hand, this phenomenon may well be the result of intracerebral administration in the mice, whereas BSE transmissibility through the oral route might be more dependent of host factors encountered in the LRS and ENS before entering the CNS [60]. However, a similar pattern of adaption has been observed in vitro by serial PMCA [60]. Overall, these results imply that passage of BSE through wt/wt and R/Q211 goats will not necessarily lead to a measurable adaptation of the BSE agent to goat. Overall, the incubation period of the wild type goats is very similar to sheep with the same *PRNP* genotype [54, 61–63].

The immunohistochemical data did not show differences between the genotypes or between the laboratories involved. More surprising are the results concerning the tissue distribution of PrP^D^. This regards both the time course of the disease and the tissues involved. Even weak PrP^D^ accumulations in peripheral tissue samples were not seen before 12 mpi and only in a single animal. Furthermore, late preclinical goats, which already have a mild to moderate accumulation of PrP^D^ in the CNS, not only showed a minor involvement of peripheral tissue samples, but in most cases even a confinement of the PrP^D^ accumulations to the Peyer’s patches and enteric nervous system of the gut. Only a few animals revealed a mild PrP^D^ positive staining reaction in other lymphoid tissues, i.e. tonsil and mesenteric lymph nodes. A more widespread distribution of PrP^D^ in lymphoid tissues was only seen in a few clinical goats, late in the incubation period. This is in clear contrast to classical scrapie in sheep and goats as well as BSE in sheep [25, 44, 62, 64]. This unusual PrP^D^ distribution pattern is reflected by examinations addressing the infectivity in peripheral tissue samples. As described by [33] and shown by results presented here none of the samples examined from goats early in the incubation period (6 and 12 mpi) revealed any signs of infectivity, neither in Tg110, Tg338 nor Tgshp IX. On the other hand similar to the results seen in immunohistochemistry a more widespread distribution of BSE infectivity was seen in different peripheral tissue samples of clinical goats among others the popliteal lymph node of goats with different genotype [33] and in the spleen of a wild type goat (lab 3, data not shown).

A French study showed that the time course of the scrapie pathogenesis is slightly prolonged in goats as compared to sheep [65]. However, the spread of the scrapie agent during the incubation time was quite similar [25, 44, 66]. This was also observed in BSE affected sheep [61, 63]. PrP^D^ accumulations are widespread with distinct PrP^D^ depositions in follicles and neurons, involving several tissues of the lymphoreticular system as well as the autonomous nervous system early in the incubation period [25, 44, 61, 63, 66]. In other words TSE neuroinvasion in sheep and goats normally did not occur until a high proportion of lymphoid tissues was positive [66]. On the other hand BSE in cattle spreads almost solely along the autonomous nervous system, with Peyer’s patches of the gut as the only lymphoid tissues regularly involved [36, 67]. Unfortunately, CMGC, as a representative sample for the autonomous nervous system, was not available for all goats. However, weak PrP^D^ accumulations were seen in two preclinical goats, distinct accumulation in one late preclinical goat as well as in all available samples of the clinical goats (data not shown). Thus, the pathogenesis seen in our BSE infected goats, with no major involvement of the lymphoreticular system is more reminiscent of the disease in cattle. This is supported by the observation that one clinical I/M142 and two late preclinical (one Q/K222 and one M/M142) goats showed no involvement of the LRS at all, even with severe PrP^D^ accumulation in the brain stem. It should be noted that the second clinical I/M142 goat revealed a slight staining reaction in rectal follicles and in tonsil. From this point, it remains a speculation what might be the reason behind this unusual distribution pattern in the LRS of BSE infections and, as described by others [66], some scrapie infected goats. There might be an inability of goat lymphoid tissues to accumulate certain PrP^D^’s. On the other hand it is also conceivable that the low amounts of PrP^D^ detectable in the LRS are due to better clearance abilities in those goats. The animal numbers here are too low to reach a conclusion on the extent to which this pattern is influenced by the genotype, but a similar pattern has been reported before [66].

The two I/M142 with clinical disease and the Q/K222 in the late preclinical state of infection showed incubation times at 44 mpi and 43 mpi, respectively. In the goat BSE challenges clinical signs were not recorded for any of the I/M142 and Q/K222 carriers, but a confirmation by IHC of PrP^D^ in the CNS of an M/M142 goat at 48 mpi and by mouse bioassay of low level infectivity in the CNS of a Q/K222 goat at 45 mpi [33] (see arrow in Figure 2) was possible. Therefore, as shown previously for goat scrapie [24, 30, 31], not only the attack ratios but also the incubation periods of BSE in goats appeared influenced by these genotypes. Low attack rates leading to few infected animals and even fewer clinical cases with long incubation periods provides valuable data in support of a protective effect against BSE infection by the M142 and K222 variants. By necessity, our study was mostly conducted in heterozygous goats and both polymorphisms, M142 and K222, showed a dominant phenotype as expected from goat scrapie studies and sheep TSE challenges [30, 31, 68]. Resistance to scrapie in the presence of either allele has been shown in many case–control studies and, due to the relative low genotype frequency of homozygotes, the association with resistance has been significant mostly for heterozygotes [15, 20, 32, 54]. The survival of the four M/M142 to 48 mpi without clinical disease suggests that homozygotes are at least as resistant to BSE as heterozygotes, which is also evident for scrapie [25]. Similar data for oral BSE exposure of K/K222 goats are still missing, but an attack ratio of 1/5 and an incubation period of 69 mpi for intracerebral inoculation with scrapie [31] combined with evidence that transgenic mice expressing only caprine K222 PrP were resistant to bovine BSE increases the likelihood that K/K222 goats will show significant resistance to BSE too [58]. Of course, this is crucial as breeding programs designed to increase the K222 allele frequency will inevitably lead to an increase in the number of homozygous K/K222 animals in goat populations. As a polymorphism that protects from scrapie and BSE, K222 is the strongest candidate for breeding programs for the eradication of TSEs from goats.

However, the K222 allele is not always available for TSE—resistance breeding, since it occurs in variable and often very low frequencies in the European goat population. It varies even within seemingly similar breeds depending on their geographical locations [17, 18, 27, 29]. Introduction of this variant into goat populations with an endemic scrapie problem may be worth considering. The use of alternative PrP polymorphisms associated with a protective effect at least for classical scrapie such as N146D and N146S may be possible for a restricted range of breeds [69, 70].

Our challenge data further strengthen the view that the caprine K222 allele is an attractive *PRNP* variant for TSE resistance breeding since it is not only strongly limiting the transmission of classical scrapie, but as is exemplified here it also has a protective effect against BSE infection.

## Additional files

**Additional file 1.** **Summary of the immunohistochemical methods, bioassays and sampling applied in the different labs involved.**

**Additional file 2.** **Additional file tables A and B: overview of goats challenged with cattle BSE (first passage) and goatBSE (second passage).**

**Additional file 3.** **Summary of the immunohistochemical results obtained from the brain stem and different peripheral tissue samples from goats infected with Cattle BSE (first passage).**

**Additional file 4.** **Summary of the immunohistochemical results obtained from the brain stem and different peripheral tissue samples from goats infected with goat BSE (second passage).**
