# Supplementary material for: Protecting effect of PrP codons M142 and K222 in goats orally challenged with bovine spongiform encephalopathy prions
Source: Vet Res. 2017 Sep 19;48:52. doi: 10.1186/s13567-017-0455-0 (PMC5606029; doi:10.1186/s13567-017-0455-0)
Supplement: Supplementary file 1 — Additional file 1. Summary of the immunohistochemical methods, bioassays and sampling applied in the different labs involved. [file 13567_2017_455_MOESM1_ESM.docx]

**Addtional file 1**

**Table:** Summary of the immunohistochemical methods, bioassays and sampling applied in the different labs involved.

|  | **lab1** | **lab2** | **lab3** |
| --- | --- | --- | --- |
| **immunohistochemistry** | | | |
| monoclonal antibody | Bar224 | 6C2, R145 | 6C2, F99 |
| antibody dilution | 1:32000 in CNS  1:16000 in LRS | 6C2: 1:50 in goat serum  R145: 1:4000 | 6C2: 1:25 in goat serum  F99: 1:4000 in goat serum |
| pretreatment | formic acid 30 min, citrate buffer pH 6.0/30min 121°C | formic acid 30 min,  citrate buffer pH 6.0/20 min 121°C | formic acid 30 min,  citrate buffer pH 6.0/20 min 121°C |
| blocking | 3% H2O2 in MethOH | 3% H2O2 in MethOH | 3% H2O2 in MethOH |
| development | N-Histofine-HRP/DAB | Vector NovaRed, Vectastain Elite ABC | Dako Envison Kit/DAB |
| biochemical analysis | | | |
| method | Western Blot | Western Blot | PTA-Immunoblot |
| material | 10% homogenate | SAF-prep | 10% homogenate |
| antibody | Sha31 | 6H4, P4 | L42, P4 |
| dilution | 1:40000 | 6H4: 0.1μg/ml  P4: 0.2 μg/ml | L42: 0.25 μg/ml  P4: 0.2 μg/ml |
| **mouse bioassays** | | | |
| mouse Line | Tg338, Tgbov110 | ND | TgshpIX |
| number of mice per sample | 6 | ND | 15 |
| mode of inoculation | 20µl i.c. | ND | 30µl i.c. |
| clinical examination | daily | ND | 2-3x/week |
| **samples** | | | |
| blood | 0, 14d, 30d, 60d, every 2 months until 24 months, then every 4 months | every 100 d | every 4 months |
| tonsil biopsies | at 12 months | every 100 d | 3 mpi to 8 mpi, excluded female goats in lactation |
| rectal biopsies | ND | ND | 3 mpi to 8 mpi, excluded female goats in lactation |

ND = Not done; d = days
