# Supplementary material for: Protecting effect of PrP codons M142 and K222 in goats orally challenged with bovine spongiform encephalopathy prions
Source: Vet Res. 2017 Sep 19;48:52. doi: 10.1186/s13567-017-0455-0 (PMC5606029; doi:10.1186/s13567-017-0455-0)
Supplement: Supplementary file 2 — Additional file 2. Additional file tables A and B: overview of goats challenged with cattle BSE (first passage) and goatBSE (second passage). [file 13567_2017_455_MOESM2_ESM.docx]

**Additional file 2**

**Additional file table A:** Overview of goats challenged with **cattle BSE (first passage)**

|  | **WT** | **IM142** | **MM142** | **RQ211** | **QK222** |
| --- | --- | --- | --- | --- | --- |
| **Negative** | n=14  (6, 15, 19, 33, 37, 38, 48 mpi) | n=8  (6, 19, 37, 48 mpi) | n=3  (48 mpi) | n=4  (6, 17 mpi) | N=10  (6, 15, 17, 25, 42 mpi) |
| **Preclinical** | n=3  (17, 19 mpi) | -- | -- | -- | -- |
| **Late Preclinical** | n=2  (19, 36 mpi) | -- | n=1  (48 mpi) | n=5  (17, 25 mpi) | n=1  (42 mpi) |
| **Clinical** | n=5  (25, 26 mpi) | n=2  (44, 45 mpi) | -- | n=3  (28, 30 33 mpi) | -- |

**Legend:** Negative = no PrP^D^/infectivity detectable; Preclinical = no PrP^D^ in CNS but in periphery (i.e. gut, lymphoid tissue, celiac and mesenteric ganglion complex, rectal enteric nervous system); Late Preclinical = PrP^D^/infectivity in brain stem but no clear clinical signs; Clinical = PrP^D^ in brain stem and clear clinical signs; mpi = moths post infection. -- No cases.

**Additional file table B:** Overview of goats challenged with **goat BSE (second passage)**

|  | **WT** | **IM142** | **MM142** | **RQ211** | **QK222** |
| --- | --- | --- | --- | --- | --- |
| **Negative** | n=16  (6, 12, 19, 25, 37, 46, 47, 81 mpi) | n=11  (6, 19, 37, 47 mpi) | ND | n=7  (6, 9, 12, 34, 77 mpi) | n=10  (6, 12, 24, 36, 44, 46, 76, 81 mpi) |
| **Preclinical** | n=2  (12, 25 mpi) | -- | ND | n=1  (12 mpi) | -- |
| **Late Preclinical** | n=3  (19, 24, 36 mpi) | -- | ND | -- | n=1  (45 mpi) |
| **Clinical** | n=5  (24-26, 28 mpi) | -- | ND | n=3  (33, 34, 36 mpi) | -- |

**Legend:** Negative = no PrP^D^/infectivity detectable; Preclinical = no PrP^D^ in CNS but in periphery (i.e. gut, lymphoid tissue, celiac and mesenteric ganglion complex, rectal enteric nervous system); Late Preclinical = PrP^D^/infectivity in brain stem but no clear clinical signs; Clinical = PrP^D^ in brain stem and clear clinical signs; mpi = months post infection; nd = not done; *infectivity in brain stem only, published by Aguilar-Calvo et al. 2015. ND = not done; -- No cases.
