# Supplementary material for: Protecting effect of PrP codons M142 and K222 in goats orally challenged with bovine spongiform encephalopathy prions
Source: Vet Res. 2017 Sep 19;48:52. doi: 10.1186/s13567-017-0455-0 (PMC5606029; doi:10.1186/s13567-017-0455-0)
Supplement: Supplementary file 3 — Additional file 3. Summary of the immunohistochemical results obtained from the brain stem and different peripheral tissue samples from goats infected with Cattle BSE (first passage). [file 13567_2017_455_MOESM3_ESM.docx]

**Addtional file 3**

**Table:**

Summary of the immunohistochemical results obtained from the brain stem and different peripheral tissue samples from goats infected with Cattle BSE (first passage).

|  | genotype | MPI | brain stem (obex) | tonsil | Ln. mes.* | ileum  (PP/ENS) | rectum  (Foll./ENS) | N. vagus | Plexus brachialis |
| --- | --- | --- | --- | --- | --- | --- | --- | --- | --- |
| preclinical | wt/wt | 17 | 0 | -- | -- | --/+ | ND | ND | ND |
|  | wt/wt | 17 | 0 | + | -- | --/-- | ND | ND | ND |
|  | wt/wt | 19 | 0 | -- | -- | --/+ | ND | ND | ND |
| late preclinical | wt/wt | 19 | 1 | -- | -- | --/-- | --/ND | ND | ND |
|  | wt/wt | 36 | 2 | + | -- | +/+ | ND/ND | -- | -- |
|  | R/Q211 | 17 | 0.5 | -- | -- | --/-- | --/-- | -- | -- |
|  | R/Q211 | 17 | 1 | -- | -- | --/+ | --/-- | -- | -- |
|  | R/Q211 | 25 | 1 | -- | -- | --/+ | --/-- | -- | -- |
|  | R/Q211 | 25 | 1 | -- | -- | --/+ | ND | -- | -- |
|  | R/Q211 | 25 | 2 | -- | + | --/+ | ND/ND | -- | -- |
|  | Q/K222** | 43 | 3 | -- | -- | --/-- | --/+ | -- | -- |
|  | M/M142 | 48 | 2 | -- | -- | --/+ | --/ND | -- | -- |
| clinical | wt/wt | 25 | 2 | -- | -- | +/+ | ND/ND | ND | ND |
|  | wt/wt | 25 | 3 | + | + | +/+ | --/+ | ND | -- |
|  | wt/wt | 25 | 3 | -- | + | +/+ | --/+ | -- | -- |
|  | wt/wt | 25 | 3 | + | -- | +/+ | --/ND | -- | -- |
|  | wt/wt | 26 | 3 | -- | + | +/+ | --/+ | -- | -- |
|  | R/Q211 | 29 | 3 | -- | + | +/+ | --/+ | -- | -- |
|  | R/Q211 | 30 | 3 | + | + | +/+ | --/+ | -- | + |
|  | R/Q211 | 33 | 3 | + | + | +/+ | --/+ | -- | -- |
|  | I/M142 | 44 | 3 | -- | -- | --/+ | ND/ND | -- | -- |
|  | I/M142 | 45 | 3 | + | -- | --/+ | --/ND | ND | ND |

**Legend:** Preclinical = no PrP^D^ in CNS but in periphery; late Preclinical = PrP^D^/infectivity in brain stem but no clear clinical signs; Clinical = PrP^D^ in brain stem and clear clinical signs; MPI = months post infection; ND = not done; + = positive result; -- = negative result; 1 = mild PrP^D^ accumulation; 2 = moderate PrP^D^ accumulation; 3 = severe PrP^D^ accumulation; *this sample includes mesenterial lymph nodes from different location of the small intestine, including the jejunum; foll. = Follicle; ENS = enteric nervous system; **this animal revealed positive results in muscles (M. psoas major and M. oculomotorius).
