# Supplementary material for: Protecting effect of PrP codons M142 and K222 in goats orally challenged with bovine spongiform encephalopathy prions
Source: Vet Res. 2017 Sep 19;48:52. doi: 10.1186/s13567-017-0455-0 (PMC5606029; doi:10.1186/s13567-017-0455-0)
Supplement: Supplementary file 4 — Additional file 4. Summary of the immunohistochemical results obtained from the brain stem and different peripheral tissue samples from goats infected with goat BSE (second passage). [file 13567_2017_455_MOESM4_ESM.docx]

**Addtional file 4**

**Table:**

Summary of the immunohistochemical results obtained from the brain stem and different peripheral tissue samples from goats infected with goat BSE (second passage).

|  | genotype | MPI | brain stem (obex) | tonsil | Ln. mes*. | ileum  (PP/ENS) | rectum  (foll./ENS) | N. vagus | Plexus brachialis | CMGC |
| --- | --- | --- | --- | --- | --- | --- | --- | --- | --- | --- |
| preclinical | wt/wt | 12 | -- | -- | -- | -- | -- | -- | -- | + |
|  | wt/wt | 25 | -- | -- | -- | -- | -- | -- | -- | + |
|  | R/Q211 | 12 | -- | -- | -- | +/-- | -- | -- | -- | -- |
| late preclinical | wt/wt | 19 | 1 | -- | -- | --/-- | --/-- | ND | ND | ND |
|  | wt/wt | 24 | 2 | -- | -- | --/+ | --/+ | -- | -- | ND |
|  | wt/wt | 36 | 1 | + | + | +/+ | -/ND | -- | -- | ND |
| clinical | wt/wt | 24 | 3 | - | - | +/+ | --/+ | -- | -- | ND |
|  | wt/wt | 25 | 3 | - | - | +/+ | --/+ | -- | -- | ND |
|  | wt/wt | 25 | 3 | - | + | +/+ | --/+ | -- | -- | ND |
|  | wt/wt | 26 | 3 | + | -- | --/+ | ++/ND | + | -- | ND |
|  | wt/wt | 28 | 3 | -- | -- | +/+ | --/ND | -- | -- | ND |
|  | R/Q211 | 33 | 3 | -- | + | +/+ | --/+ | -- | -- | ND |
|  | R/Q211 | 34 | 3 | -- | -- | --/+ | --/+ | -- | -- | ND |
|  | R/Q211 | 36 | 2 | -- | -- | --/+ | --/+ | -- | -- | ND |

**Legend:** Preclinical = no PrP^D^ in CNS but in periphery; late preclinical = PrPD/infectivity in brain stem but no clear clinical signs; clinical = PrP^D^ in brain stem and clear clinical signs; MPI = months post infection; ND = not done; + = positive result; -- = negative result; 1 = mild PrP^D^ accumulation; 2 = moderate PrP^D^ accumulation; 3 = severe PrP^D^ accumulation; *this sample includes mesenterial lymph nodes from different location of the small intestine, including the jejunum; Foll. = Follicle; ENS = enteric nervous system; CMGC = celiac and mesenteric ganglion complex.
